# Formaldehyde Exposure Racial Disparities in Southeast Texas

**DOI:** 10.1021/acs.est.3c02282

**Published:** 2024-02-27

**Authors:** Yiting Li, Yusheng Zhao, Michael J. Kleeman

**Affiliations:** † Department of Civil and Environmental Engineering, University of California, Davis, California 95616, United States; ‡ Department of Land, Air, and Water Resources, University of California, Davis, California 95616, United States

**Keywords:** exposure disparity, cancer risk, source apportionment, air pollution

## Abstract

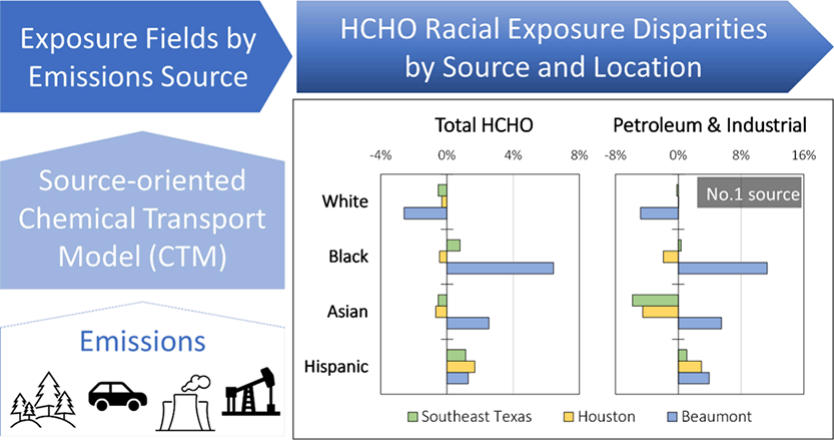

Formaldehyde (HCHO) exposures during a full year were calculated for different race/ethnicity groups living in Southeast Texas using a chemical transport model tagged to track nine emission categories. Petroleum and industrial emissions were the largest anthropogenic sources of HCHO exposure in Southeast Texas, accounting for 44% of the total HCHO population exposure. Approximately 50% of the HCHO exposures associated with petroleum and industrial sources were directly emitted (primary), while the other 50% formed in the atmosphere (secondary) from precursor emissions of reactive compounds such as ethylene and propylene. Biogenic emissions also formed secondary HCHO that accounted for 11% of the total population-weighted exposure across the study domain. Off-road equipment contributed 3.7% to total population-weighted exposure in Houston, while natural gas combustion contributed 5% in Beaumont. Mobile sources accounted for 3.7% of the total HCHO population exposure, with less than 10% secondary contribution. Exposure disparity patterns changed with the location. Hispanic and Latino residents were exposed to HCHO concentrations +1.75% above average in Houston due to petroleum and industrial sources and natural gas sources. Black and African American residents in Beaumont were exposed to HCHO concentrations +7% above average due to petroleum and industrial sources, off-road equipment, and food cooking. Asian residents in Beaumont were exposed to HCHO concentrations that were +2.5% above average due to HCHO associated with petroleum and industrial sources, off-road vehicles, and food cooking. White residents were exposed to below average HCHO concentrations in all domains because their homes were located further from primary HCHO emission sources. Given the unique features of the exposure disparities in each region, tailored solutions should be developed by local stakeholders. Potential options to consider in the development of those solutions include modifying processes to reduce emissions, installing control equipment to capture emissions, or increasing the distance between industrial sources and residential neighborhoods.

## Abstract 2

Primary formaldehyde emissions and its precursors from industrial facilities add to regional secondary formaldehyde concentrations and contribute strongly to exposure disparities by race/ethnicity in Southeast Texas.

## 1 Introduction

Formaldehyde (HCHO) is a ubiquitous organic compound found in urban atmospheres across the globe.^1,2^ HCHO concentrations in urban areas are typically an order of magnitude higher than concentrations of larger aldehydes such as acetaldehyde or more complex molecules with an aldehyde functional group.^3^ HCHO can be emitted directly to the atmosphere (primary), or it can form as a product from the reaction of more complex organic molecules (secondary). Once formed, HCHO goes on to further react with oxidants such as hydroxyl radicals (OH) or to photolyze in the presence of sunlight, leading to radicals that contribute to ozone formation.^4−7^

Many epidemiology studies have demonstrated a positive relationship between HCHO exposure and cancer risk.^8−12^ The World Health Organization (WHO) classified HCHO as a human carcinogen in the year 2004.^13,14^ HCHO was ranked as the greatest cancer driver of risk across the United States by the US Environmental Protection Agency in the year 2018.^15^ Exposure to HCHO represents a continuing public health risk that must be understood before it can be efficiently mitigated.

Daily satellite measurements have found that HCHO concentrations follow a spatial distribution similar to biogenic sources in the US, implying that biogenic emissions are a major source of HCHO. Biogenic sources emit isoprene that reacts in the atmosphere to form HCHO.^16−19^ More than 90% of the HCHO associated with biogenic sources forms through this secondary reaction pathway.^16,17,20−23^ Identification of other HCHO sources using satellites is challenging. Most satellite studies average concentrations over weeks or months in order to remove the noise in the measurements. The spatial resolution of older satellite observations is also somewhat coarse (13 km × 24 km), making it difficult to identify the effects of point sources.^24^ Ground-based measurement campaigns such as TexAQS in 2000,^25−29^ TexAQS II in 2006,^30−32^ and SHARP in 2009^5,33^ measured HCHO with a higher temporal and spatial resolution. The results from these campaigns suggest that significant primary HCHO concentrations may be present near industrial facilities in addition to widespread “background” concentrations of secondary HCHO. Recent studies in other industrialized countries also show that primary HCHO may be significant, accounting for as much as 70% of the total HCHO in the regions adjacent to industrial facilities.^34−37^

Historical housing practices such as “redlining” and structural income inequities have established racially segregated neighborhoods in cities across the US.^38,39^ Minority neighborhoods are often located near industrial facilities or transportation corridors that have higher levels of air pollution.^40,41^ Past studies have explored exposure disparities as a function of race/ethnicity for PM_2.5_ mass,^42,43^ PM_0.1_ mass,^44,45^ ozone,^43^ and nitrogen dioxide.^44,46^ Fewer studies^47^ have explored disparities for HCHO exposures, and none of these prior studies quantified HCHO exposure disparities and combined the analysis with apportionment calculations to identify the sources of HCHO exposure.

Source apportionment of outdoor HCHO concentrations is difficult due to the complexity of primary and secondary production routes and atmospheric reactivity. Specialized models have been developed in previous studies to quantify source contributions to other photochemical pollutants including ozone (O_3_).^48−54^ Here, we adapt the methods used for O_3_ source apportionment calculations to track sources of primary and secondary HCHO production during the year 2017 in southern Texas. Total population exposure calculations are performed for 10 different formaldehyde sources. Exposure disparities by race/ethnicity are calculated, and preliminary strategies to reduce these disparities are presented.

## 2 Methods

### 2.1 Chemical Transport Model with Tagging

Air quality simulations were conducted over the southern US for the year 2017 using the UCD/CIT chemical transport model (CTM)^55^ with extensions for the source apportionment of O_3_ and other photochemical species.^51^ Year 2017 was selected for analysis as the most recent annual period with a published version of the National Emissions Inventory (NEI) prior to the COVID-19 pandemic. Simulations were conducted using a parent 24 km domain, which covers most of Texas and part of Louisiana followed by a nested 4 km domain covering Southeast Texas (see Figure S1). The UCD/CIT airshed model is a reactive 3D chemical transport model (CTM) that predicts the evolution of gas and particle phase pollutants in the atmosphere in the presence of emissions, transport, deposition, chemical reaction, and phase change. The basic capabilities of the UCD/CIT model are similar to the Community Multiscale Air Quality Modeling System (CMAQ) maintained by the US EPA, but the UCD/CIT model has additional source apportionment features and higher particle size resolution.

Source apportionment calculations for HCHO within the UCD/CIT model are accomplished using “tagging” that divides pollutants emitted from different sources into predefined groups. Pollutants within each group react to form products that also belong to that group. For example, methanol (CH_2_OH) reacts with hydroxyl radicals to produce HCHO. Tagging keeps track of the sources that emit CH_2_OH and associates any HCHO that forms with the original source.
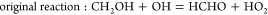

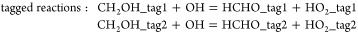


The tagging procedure is an accounting exercise that follows the source identity of pollutants through the photochemical reaction mechanism without altering the rate of reaction or the total amount of each pollutant. NEI emission inventories were reported by Source Classification Codes (SCC). Emissions in the current study were organized and tagged into nine separate groups based on their expected contributions to HCHO concentrations: (1) type 1, on-road gasoline mobile; (2) type 2, petroleum and industrial; (3) type 3, on-road diesel mobile; (4) type 4, off-road gasoline and diesel equipment; (5) type 5, residential wood combustion; (6) type 6, food cooking and on-road CNG, E85; (7) type 7, natural gas combustion; (8) type 8, biogenic; (9) type 9, aircraft and other emissions not included in the categories listed above. Detailed SCCs included in each type are shown in Tables S1−S5. Initial and boundary conditions (ICBCs) from the MOZART global chemistry model were tagged as a “tenth source” and tracked separately through the UCD/CIT simulations. Species from each tagged category described above were followed through the SAPRC11 chemical reaction mechanism.

Two air quality simulations were used in the current study: (1) w/chem, with chemical reactions turned on to track primary and secondary source contributions to HCHO, and (2) w/o chem, with chemical reactions turned off to track primary source contributions to HCHO. Population-weighted concentrations (PWCs) for HCHO were calculated in both simulations to quantify exposure to sources of primary and secondary HCHO experienced by the average person in the study domain. Equation 1 defines the PWC:
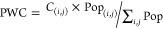
1where *C*_(*i*,*j*)_ is the model-estimated concentration in the grid cell (*i*,*j*); Pop_(*i*,*j*)_ is the population in the grid cell (*i*,*j*); ∑_*i*,*j*_Pop is the total population in the selected study region.

### 2.2 Model Inputs

Emissions were generated using the Sparse Matrix Operator Kernel Emissions (SMOKE v4.7) modeling system applied to the 2017 National Emissions Inventory (NEI). Biogenic emissions for the year 2017 were included in source type 8 based on the Model of Emissions of Gases and Aerosols from Nature (MEGAN v2.1).^56^

Meteorology data used to drive the MEGAN v2.1 biogenic emission model and the UCD/CIT CTM were generated using the Weather Research and Forecasting model (WRF v4.3). Meteorological fields were created within three nested domains with horizontal resolutions of 36, 12, and 4 km. Each domain had 31 telescoping vertical levels up to a top height of 12 km. Four-dimensional data assimilation (FDDA) or “FDDA nudging” was used to anchor meteorological predictions to measured values. A comparison between model-estimated and measured temperature and wind speed is shown in Figure S2.

### 2.3 Environmental Justice Analysis

Spatially resolved CTM pollution fields were combined with race/ethnicity data from the 2017 American Community Survey (ACS) data set (https://www2.census.gov/geo/tiger/TIGER_DP/). HCHO exposure for “Asian alone”, “White alone”, “Black and African American”, and “Hispanic or Latino, regardless of race” was calculated based on home address information aggregated in the ACS data set. The relative disparity in HCHO exposure for each race/ethnicity group was calculated by comparison to the population-weighted HCHO exposure.

Environmental Justice (EJ) analysis was performed for the entire simulated domain and for two subregions in Southeast Texas (Houston−Harris County and Beaumont and Port Arthur−Jefferson County) in order to better understand localized exposure patterns that may be influenced by concentration hot spots (see Figure S1). Harris and Jefferson have been listed previously among the top 19 counties that have high HCHO adverse health outcomes across the US.^47^ The Beaumont and Port Arthur EJ region will be referred to as Beaumont in the following sections.

## 3 Results

### 3.1 Characteristics of the HCHO Concentration Field and Comparison to Measurements

**Figure 1:**
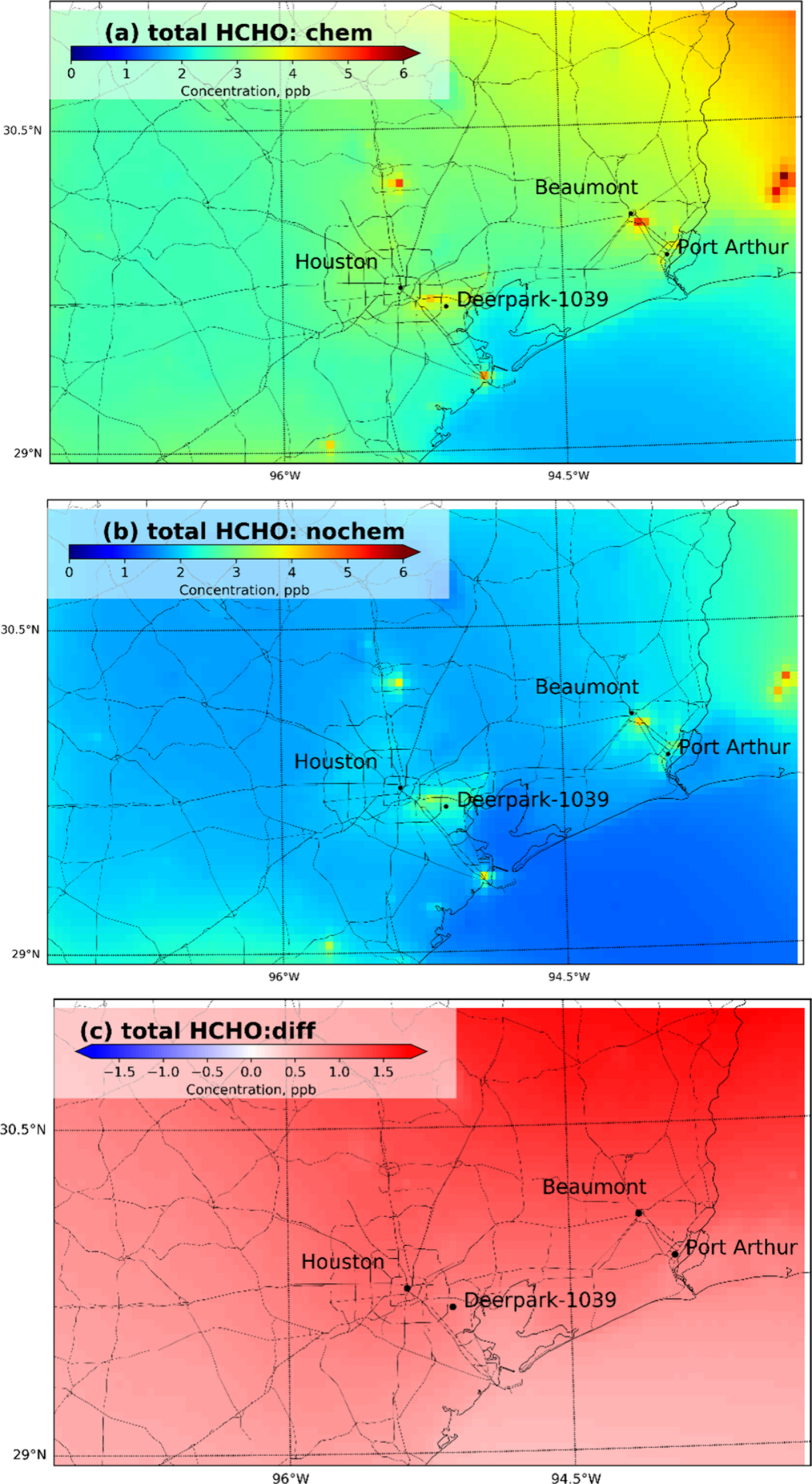
Year 2017 estimated HCHO concentration field in Southeast Texas: (a) HCHO concentrations with chemical reactions turned on, (b) HCHO concentrations without chemical reactions, and (c) secondary HCHO concentration field = difference between (a) and (b).

Figure 1 shows the HCHO concentration field averaged during the year 2017, with chemical reactions turned on (w/chem, Figure 1a) and with chemical reactions turned off (w/o chem, Figure 1b). Figure 1c shows the concentration difference between the w/chem case and the w/o chem case to quantify the secondary production of HCHO. Note that Figure 1c quantifies secondary HCHO produced within the UCD/CIT model domain, but it does not differentiate between primary and secondary background HCHO that enters the model domain as boundary conditions. This background HCHO will be discussed separately in the analysis.

HCHO concentrations are 1 to 6 ppb across most of the study region. The spatial patterns of the maximum HCHO concentrations estimated in the w/chem and w/o chem simulations are similar. Figure 1c shows that secondary production adds approximately 1.5 ppb to HCHO concentrations across the study domain. These findings suggest that secondary production contributes significantly to “baseline” exposure for the majority of the study population, but primary emissions of HCHO or reactive HCHO precursors drive the concentration hot spots in the total HCHO exposure field.

**Figure 2:**
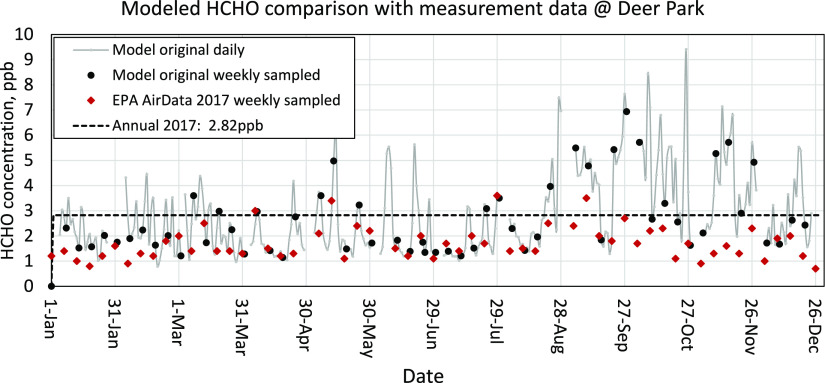
Estimated HCHO concentration comparison with EPA measurement−site 482011039, Deer Park, near Houston Ship Channel. Black dots correspond to daily average estimated concentrations for comparison to measurements (red diamonds).

Estimated HCHO concentrations during 2017 were compared with HCHO measurements at the Deer Park site maintained by US EPA (Figure 2). Deer Park is located south of the Houston Ship Channel and is surrounded by many industrial and petroleum facilities. The gray line in Figure 2 represents daily estimated HCHO concentrations, and the black dots emphasize the estimated concentrations on days with measurements (red diamonds). Model predictions are generally in good agreement with measured concentrations from mid-Jan through Jul, but estimated concentrations are higher than measured concentrations during the fall and winter months. These overpredictions are driven by seasonal changes in the underlying emission inventory. Total HCHO emissions during October are 33% (summer) to 50% (winter) higher than other months, largely due to increased emissions from petroleum and industrial sources. Emission rates should be directly measured at multiple refineries to verify the HCHO seasonal profile used in the NEI. Also, two major point sources are located in grid cells adjacent to the measurement site (Figure S11), making Deer Park particularly responsive to primary emissions from this sector. Increased spatial resolution for model calculations could partially mitigate this effect by better resolving sharp spatial gradients around point source emissions.^57^ The trends illustrated in Figure 2 reflect common issues when comparing gridded model and point measurement data.

Estimated annual HCHO concentrations at Deer Park were also compared to one available measurement site, Cloverleaf maintained by the Houston Health Department in 2019. Annual average concentrations are expected to be similar in adjacent years. Cloverleaf is located to the north of the Houston Ship Channel (Figure S11).^58^ The measured annual average HCHO concentration of 2.28 ppb at Cloverleaf compares favorably to the estimated annual average HCHO concentration of 2.35 ppb. The annual average is 16 times higher than EPA’s chronic health screening level of 0.17 ppb.^59^

**Figure 3:**
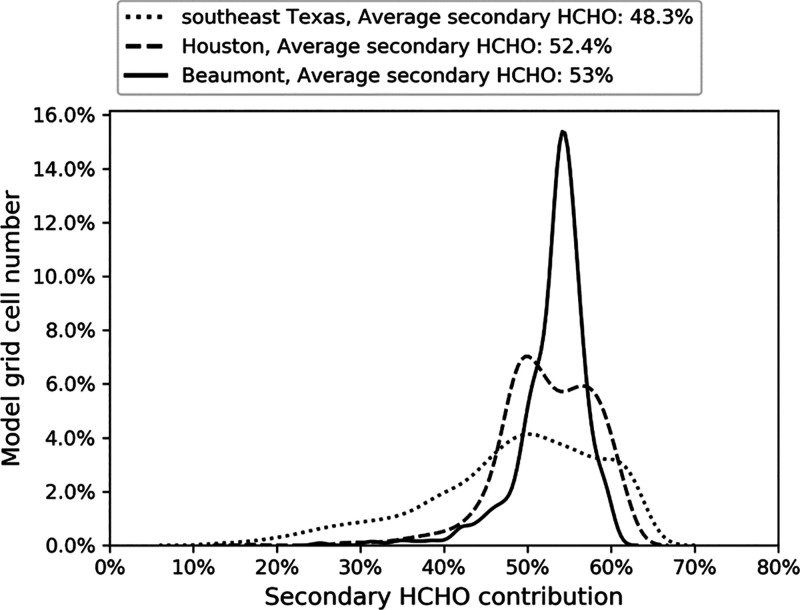
Year 2017 annual HCHO secondary contribution. Does not include background HCHO.

Figure 3 shows that approximately 45−60% of the annual average ground-level HCHO in each of the three UCD/CIT analysis domains is produced by secondary reactions. The remaining HCHO is produced by primary emissions that are more likely to generate sharp spatial gradients that may contribute to exposure disparities. Several previous studies have estimated secondary HCHO contributions in the Houston area. Friedfeld et al.^60^ attributed 63% of HCHO to secondary production and 37% to primary sources. Buzcu Guven and Olaguer^30^ attributed 60% of HCHO to secondary production and 40% to primary sources. Rappenglück et al.^61^ attributed 24% of HCHO to secondary production, 47% to primary sources, and 29% unknown. Each of these results was based on ambient measurements collected during te summer season. Green et al.^62^ found that secondary HCHO is higher in the summer and lower in the winter. Parrish et al.^63^ estimated a much higher HCHO secondary production of 92%, but these estimates are derived for the entire atmospheric column throughout the region, not for the ground-level sites along the Houston Ship Channel. Secondary HCHO contributions estimated by previous studies therefore range from 24 to 92%. Applying this split to the boundary conditions in the current study and combining them with direct concentrations within the UCD/CIT model domains that are summarized in Figure 3 yield an estimated secondary HCHO contribution of 41−62% across the study region. This estimated range is consistent with the central tendency of the results produced by previous studies,^16,20^ building confidence in the accuracy of the model calculations.

It should be noted that the population-weighted exposure concentration (PWC) depends strongly on the population distribution in the study region (Figure S3). Houston is the largest population center followed by Beaumont and Port Arthur to the east. The high HCHO concentrations in Beaumont have a moderate impact on PWC across the entire study region because the population in Beaumont is significantly lower than the population in the Houston region (Tables 1 and 2). Separate impacts for HCHO exposures in the region around Houston and the region around Beaumont Area are presented in the following sections.

### 3.2 Source Apportionment

Table 1 summarizes the annual PWC for each HCHO source in three regions: (i) Southeast Texas, (ii) the area around Houston (Harris County), and (iii) the area around Beaumont (Jefferson County). HCHO sources are listed in order of decreasing PWC in Southeast Texas. Exposure concentrations are estimated in both the w/chem simulation and the w/o chem simulation. Secondary HCHO concentrations were estimated by subtracting w/o chem PWC from the w/chem PWC. As noted previously, results for Southeast Texas are dominated by the large population in the area around Houston.

**Table 1:** Source Apportionment Analysis in Three Study Regions^a^

total PWC	3.013 ppb		3.402 ppb		3.010 ppb	
region	Houston		Beaumont		Southeast Texas	
source	PWC w/chem	secondary PWC %	PWC w/chem	secondary PWC %	PWC w/chem	secondary PWC %
petroleum and industry	1.300	48.23%	1.835	45.07%	1.337	47.35%
biogenic	0.338	91.99%	0.353	91.11%	0.351	91.80%
others and aircraft	0.153	95.23%	0.043	98.45%	0.134	95.66%
natural gas combustion	0.078	−57.56%	0.173	−30.00%	0.084	−53.83%
off-road equipment	0.112	−0.16%	0.049	−7.13%	0.095	−0.63%
						
on-road gasoline	0.076	10.45%	0.018	15.93%	0.063	11.47%
on-road diesel	0.059	0.34%	0.022	0.95%	0.049	0.39%
						
residential combustion	0.004	−5.41%	0.002	−9.00%	0.003	−5.77%
food cooking and CNG, E85	0.001	4.19%	0.001	6.83%	0.001	4.63%
						
ICBCs	0.892	-	0.906	-	0.891	-

a “PWC w/chem” shows the population-weighted HCHO concentration in the domain (what the average person experienced), units in ppb; “total PWC” in the first row is the sum of PWC across all source types; “secondary PWC %” is the estimated contribution from chemical reactions rather than primary emissions. Negative “secondary PWC %” indicates that the chemical reactions consume more primary HCHO than they produce.

**Figure 4:**
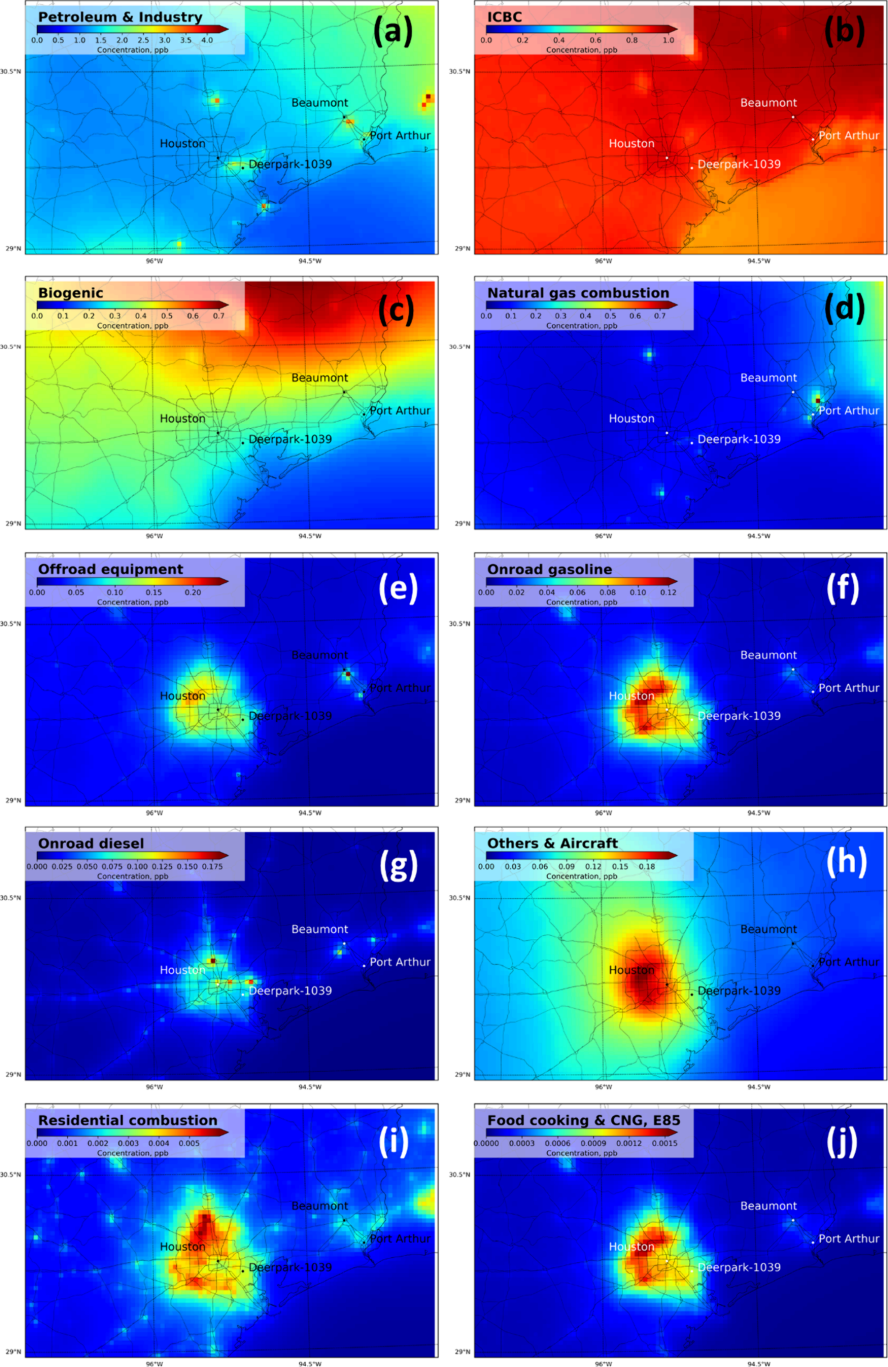
(a−j) HCHO concentration fields with chemical reactions (primary + secondary) for top 9 HCHO sources and background.

Figure 4 shows HCHO concentration fields for all source types and background in Southeast Texas averaged over the year 2017. Petroleum and industrial sources, natural gas combustion, and off-road diesel sources produce HCHO hot spots that may contribute to exposure disparities, but the affected regions and relative magnitudes of these sources are not equivalent. The petroleum and industrial category (Figure 4a) is the largest anthropogenic source of HCHO, contributing 44% of the total HCHO PWC in Southern Texas, 43% of the total HCHO PWC in the region around Houston, and 53% of the total HCHO PWC in the regions around Beaumont (Table 1). The spatial pattern of petroleum and industrial HCHO dominates the total HCHO field (compare Figure 1a to Figure 4a). An analysis of the w/o chem simulations shows that ∼55% of the HCHO associated with petroleum and industrial sources is primary (Table 1). HCHO hot spots associated with this source occur along the Houston Ship Channel and near the city of Beaumont. The maximum annual average HCHO concentration in Jefferson County is estimated to be 3.5 ppb, and the maximum annual average HCHO concentration in Harris County is estimated to be 2.5 ppb. These values are 20 and 15 times higher than the EPA chronic health screening level.^59^

Petroleum and industrial facilities also emit HCHO precursors such as ethylene and propylene (see Figure S12) that quickly react to form secondary HCHO around the emission location. Secondary HCHO production can also contribute to sharp spatial gradients in HCHO concentrations under these conditions, potentially exacerbating exposure disparities.

Biogenic sources in Southern Texas (Figure 4c) account for 11% of the total HCHO PWC averaged across the entire year (Table 1). Biogenic sources emit isoprene that reacts in the atmosphere to form HCHO. Isoprene emissions have a strong seasonal trend, with higher rates in summer and lower rates in winter.^64^ As a consequence, the biogenic secondary HCHO PWC ranges from 3% in the winter to 25% in the summer, making biogenic emission sources a dominant HCHO source or a minor HCHO source depending on the season (Figure S9). It is worth noting that interannual temperature variability can significantly affect isoprene emissions.^16^ Increasing temperature consistent with climate change would be expected to increase biogenic contributions to HCHO concentrations.

Other and aircraft sources (Figure 4h) are the third largest source of population-weighted HCHO exposure, contributing 4.5% to total PWC in Southern Texas (Table 1). Approximately 95% of the HCHO associated with other sources is produced by secondary reactions (Table 1), and the minor primary HCHO emissions in this category are associated with area sources that do not produce sharp spatial gradients in HCHO concentrations.

Off-road equipment sources (Figure 4e) and natural gas combustion (Figure 4d) both emit primary HCHO that reacts in the atmosphere. Off-road equipment contributes 3.7% to HCHO concentrations in Houston, while natural gas combustion contributes 5% to HCHO concentrations in Beaumont. Both sources exhibit concentration hot spots that may contribute to localized exposure disparities.

The spatial pattern of HCHO associated with on-road gasoline vehicles (Figure 4f) and on-road diesel vehicles (Figure 4g) mirrors the population density across the study region. On-road diesel vehicles have additional peaks at the Houston International Airport and the Houston Ship Channel due to goods movement activities. Peak concentrations of HCHO associated with mobile sources are not dominant across the region, but the alignment with population results in significant exposures. Chemical reactions contribute to HCHO from mobile sources, with 11% secondary HCHO production from on-road gasoline vehicles and 0.4% secondary HCHO production from on-road diesel vehicles (Table 1).

### 3.3 Environmental Justice (EJ) Analysis

Separate EJ analyses were performed in the region around Houston, the region around Beaumont, and Southeast Texas to better quantify the different source contributions to exposure disparities in each location. The total population and percentage of each race/ethnicity in Southeast Texas, Houston, and Beaumont are summarized in Table 2. Population distributions for White alone, Black and African American, Asian alone, and Hispanic or Latino residents are shown in Figures S4−S7 in the SI. White residents account for 65% the total population in Southeast Texas, with most white residents living around Houston. Black residents account for 17% of the total population, with most Black residents living in the outlying suburbs of Houston. A smaller subset of the Black population lives in Beaumont. Asian residents account for 5.4% of the total population, with most Asian residents living west of Houston, especially southwest of Houston. Hispanic residents account for 32% of the total population, with the majority of the Hispanic residents living on the east side of Houston, especially near the Ship Channel.

**Figure 5:**
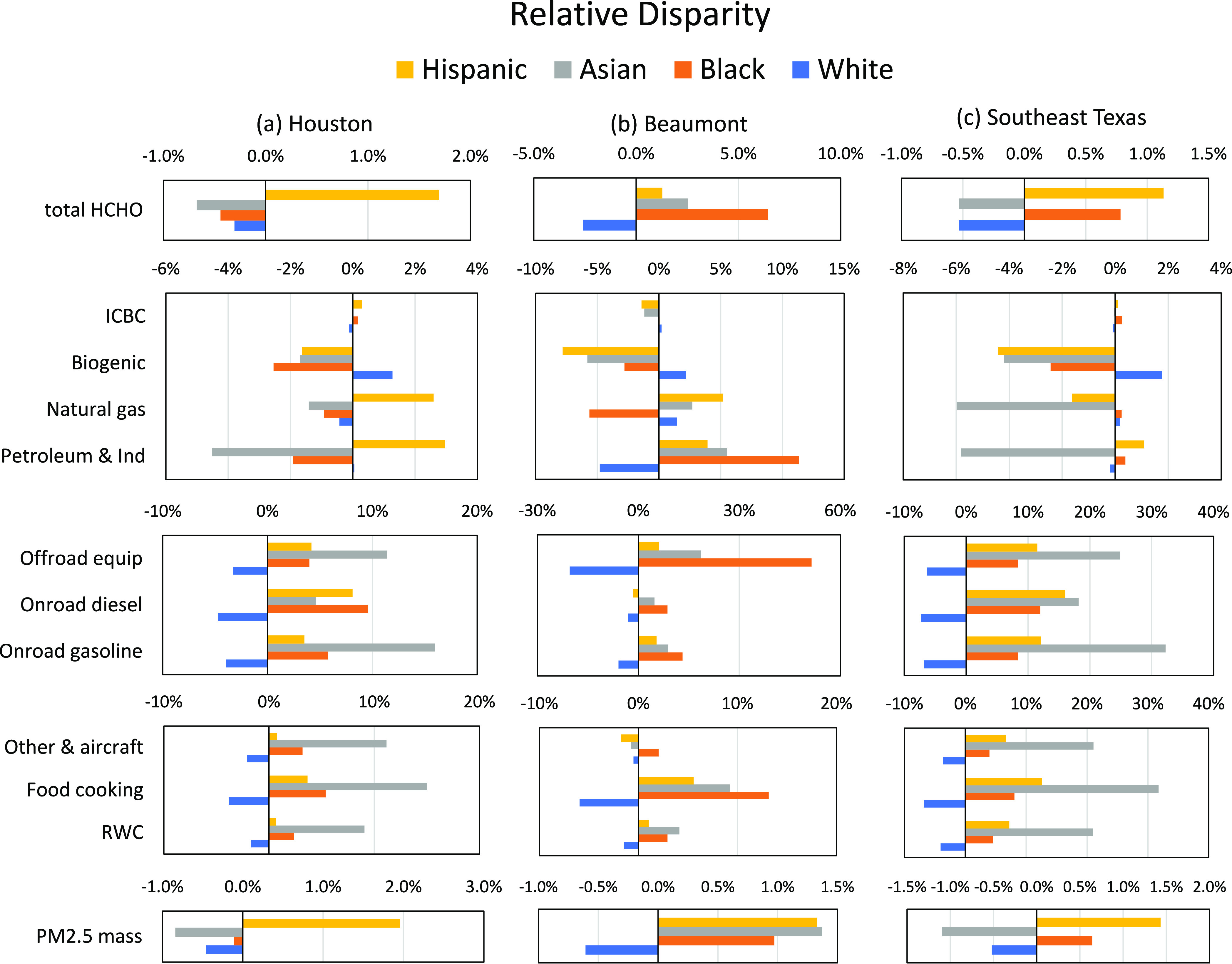
HCHO Relative disparity by race/ethnicity in three study regions based on year 2017 annual PWC: (a) Houston, (b) Beaumont, and (c) Southeast Texas for 9 HCHO sources, total HCHO, and PM_2.5_ total mass. RWC represents residential wood combustions.

Figure 5 summarizes the results of HCHO exposure calculations stratified by race/ethnicity in Houston (Figure 5a), Beaumont (Figure 5b), and Southeast Texas (Figure 5c). The PM_2.5_ mass is included in the analysis since this pollutant carries the greatest risk to increased all-cause mortality, while HCHO carries the greatest risk for cancer. White residents are consistently exposed to 0−5% below average HCHO concentration for all sources except for biogenic, as well as total HCHO and PM_2.5_ mass in the three study regions.

The rank of groups with above average HCHO exposure are different for Houston and Beaumont. Hispanic residents are the highest exposure group in Houston (Figure 5a) for major HCHO sources (petroleum and industrial) as well as total HCHO and PM_2.5_ mass (2−3% above average). This exposure pattern is related to the high population density of Hispanic residents along the Houston Ship Channel (Figure S6). Asian residents are exposed to below average total HCHO concentrations in Southeast Texas and Houston and 2.5% above average total HCHO exposure in Beaumont.

Above average HCHO exposures in Beaumont are associated with petroleum and industrial sources, off-road equipment, and food cooking. Black residents in Beaumont (Figure 5b) are exposed to total HCHO concentrations that are 7% above average. The largest anthropogenic sources of HCHO exposure for Black residents are petroleum and industrial facilities. These emissions are clustered near the refineries present in Beaumont (Figure S5). Hispanic residents are the second highest HCHO exposure group in Beaumont due to primary HCHO emissions near Port Arthur. Thus, the total HCHO disparity in Beaumont reflects a combination of effects associated with petroleum and industrial sources and natural gas combustion.

Total HCHO exposure disparities across Houston and Beaumont combine to produce a 1% above average HCHO exposure for Hispanic and Black residents in Southeast Texas and a 0.5% below average HCHO exposure for Asian and White residents. Petroleum and industrial sources contribute most strongly to these disparities. Disparities in the smaller population centers can be larger than disparities across the entire region, emphasizing the need for localized analysis and solutions. Disparities across the entire region are dominated by common sources such as on-road gasoline, on-road diesel, off-road equipment, and other and aircraft, which are generally related to human activities, not industrial processes. The diverse sources that contribute to HCHO exposure disparities will require coordinated control strategies across multiple emission sectors.

**Table 2:** Race/Ethnicity Population Data Summary

	Southeast Texas		Houston		Beaumont	
race	population	percentage	population	percentage	population	percentage
White	4,151,047	65.48%	3,085,297	62.69%	221,627	65.62%
Black	1,093,732	17.25%	878,721	17.86%	81,828	24.23%
Asian	345,158	5.44%	318,057	6.46%	9167	2.71%
Hispanic	2,054,893	32.42%	1,774,908	36.07%	43,336	12.83%
Total	6,339,318		4,921,248		337,737	

## 4 Discussion

The greatest disparities in HCHO exposures in Southeast Texas identified in the current study are driven by local industrial sources. Each source has unique characteristics that require individual analysis. Previous EJ studies focused on PM_2.5_ also found exposure disparities with unique regional features in Portland, Oregon, in Salem, Oregon,^65^ in Los Angeles, California, and in the San Francisco Bay Area, California.^45^ It is unlikely that a universal solution exists to mitigate these local exposure disparities, but some general principles can be considered while developing tailored solutions. As with any EJ discussion, the full development of solutions should involve all stakeholders, especially the affected community. Potential mitigation options include changing the industrial process to reduce the harmful emissions, installing control devices to capture harmful emissions prior to release, or increasing the distance between the industrial facility and the residential neighborhoods where exposure occurs. Each of these options involves economic and social trade-offs that are beyond the scope of the current study to analyze. The detailed exposure analysis included here will help provide information to support the process of developing appropriate solutions.

The EPA chronic health screening level for HCHO is set to be 0.17 ppb to reduce the risk for additional cancer cases below one per million people. The population-weighted outdoor concentrations estimated in the current study exceed this screening level by more than a factor of 10, emphasizing the importance of understanding outdoor HCHO sources and formation pathways to protect public health in Southeast Texas. HCHO concentrations in the indoor environment are often significantly higher than outdoor concentrations due to a number of potential indoor sources^66−68^ and relatively low indoor dilution rates.^69^ Outdoor HCHO concentrations still affect public health because outdoor HCHO provides a significant background that increases indoor exposures.^70^

The current exploratory study using models helps to identify potential HCHO hot-spot locations that may be difficult to observe using satellites with a limited spatial resolution. Ground-based measurements will be able to confirm these estimated HCHO concentrations. Previous field campaigns that made ground-based measurements in Houston detected HCHO concentrations that exceeded 50 ppbv.^71^ Further analysis of those measured values determined that these high HCHO concentrations were associated with primary emissions from industrial facilities.^71^ The model-estimated concentration pattern in Beaumont follows this same trend. The results further emphasize the utility of CTMs to identify exposure hot spots that can be further investigated and confirmed with direct measurements. This combined approach will either confirm the existence of HCHO hot spots or, alternatively, identify a problem with the HCHO emission inventory that must be corrected in order to more accurately assess public health risk.

## Supporting Information Available

Figures and tables illustrating the model domain configuration, population distributions, time series of formaldehyde exposures, spatial maps of formaldehyde exposures, spatial maps of formaldehyde precursors, and listings of Emissions Inventory Codes (EICs) that release formaldehyde (PDF)
