# Supplementary material for: Formaldehyde Exposure Racial Disparities in Southeast Texas
Source: Environ Sci Technol. 2024 Feb 27;58(10):4680–90. doi: 10.1021/acs.est.3c02282 (PMC10938643; doi:10.1021/acs.est.3c02282)
Supplement: Supplementary file 1 — es3c02282_si_001.pdf [file es3c02282_si_001.pdf]

Formaldehyde exposure racial disparities in southeast Texas

Supporting Information

Yiting Li[1], Yusheng Zhao[2], and Michael J. Kleeman[1, *]

[1]Department of Civil and Environmental Engineering, University of California, Davis, 95616, United States

[2]Department of Land, Air, and Water Resources, University of California, Davis, 95616, United States

E-mail address:

Yiting Li: yitli@ucdavis.edu

Yusheng Zhao: yshzhao@ucdavis.edu

Michael J. Kleeman: mjkleeman@ucdavis.edu

Page 19, Figure 12, Table 5

Figure S 1. Chemical transport model and Enviornmental Justice (EJ) domain settings. 2
Figure S 2. WRF temperature and wind speed compaison to EPA measurement at site 1039 – deer park.2
Figure S 3. Total population spatial distribution in target area. 3
Figure S 4. Asian alone population density in Houston – Beaumont area...4
Figure S 5. Black & African American alone population density in Houston – Beaumont area...................5
Figure S 6. Hispanic or Latino, regardless of races population density in Houston – Beaumont area. 6
Figure S 7. White alone population density in Houston – Beaumont area..7
Figure S 8. Monthly HCHO population weighted concentration over Houston EJ domain, Beaumont EJ domain, and Southeast Texas for w/ chem simulation and w/o chem simulation. 8
Figure S 9. Monthly HCHO population weighted concentration % over Houston EJ domain, Beaumont EJ domain, and Southeast Texas for w/ chem simulation and w/o chem simulation. 9
Figure S 10. Secondary HCHO contributions...10
Figure S 11. Measurement sites geospatial information...11
Figure S 12. HCHO precursors emissions – Ethylene and Propylene..12

Table S 1. Source type – Petroleum & Industrial top 20 SCC for months except for October...................13
Table S 2. Source type – Petroleum & Industrial top 20 SCC for October only. 14
Table S 3. Source type – Offroad equipment top 20 SCC. 15
Table S 4. Source type – natural gas combustion top 19 SCC..16
Table S 5. Source type – other & aircraft top 11 SCC. 17

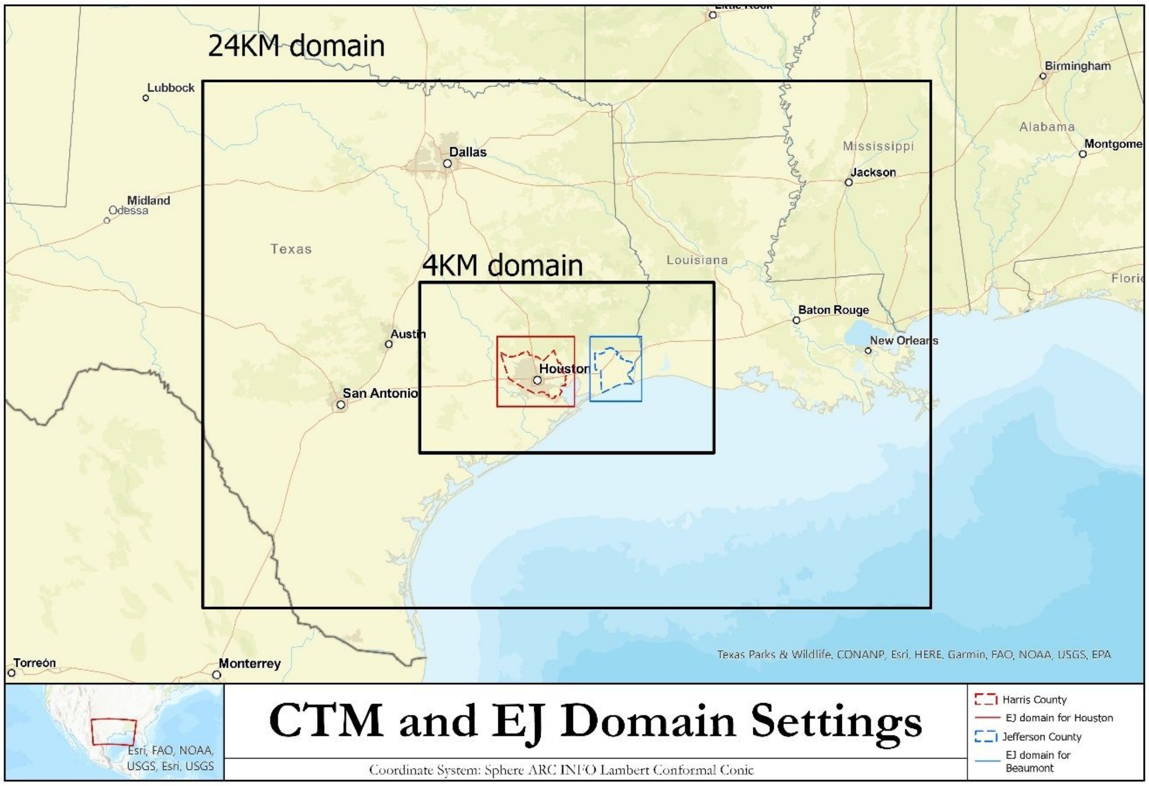

Figure S 1. Chemical transport model and Enviornmental Justice (EJ) domain settings. Figure created by ArcGIS, licensed by UC Davis.

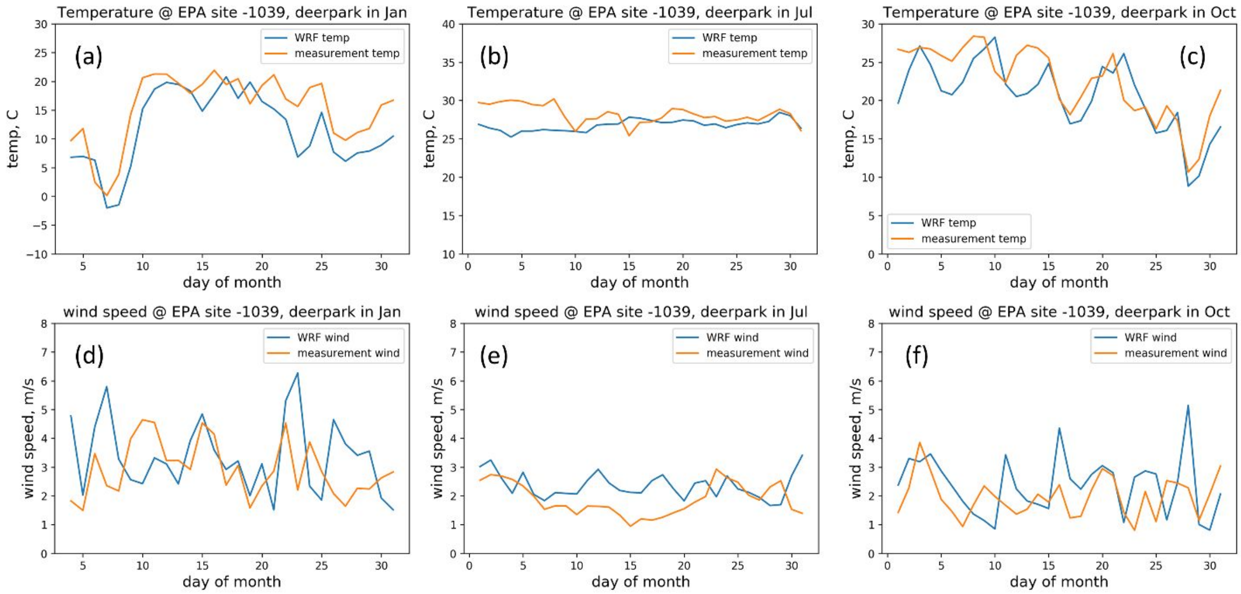

Figure S 2. WRF temperature and wind speed compaison to EPA measurement at site 1039 – deer park.

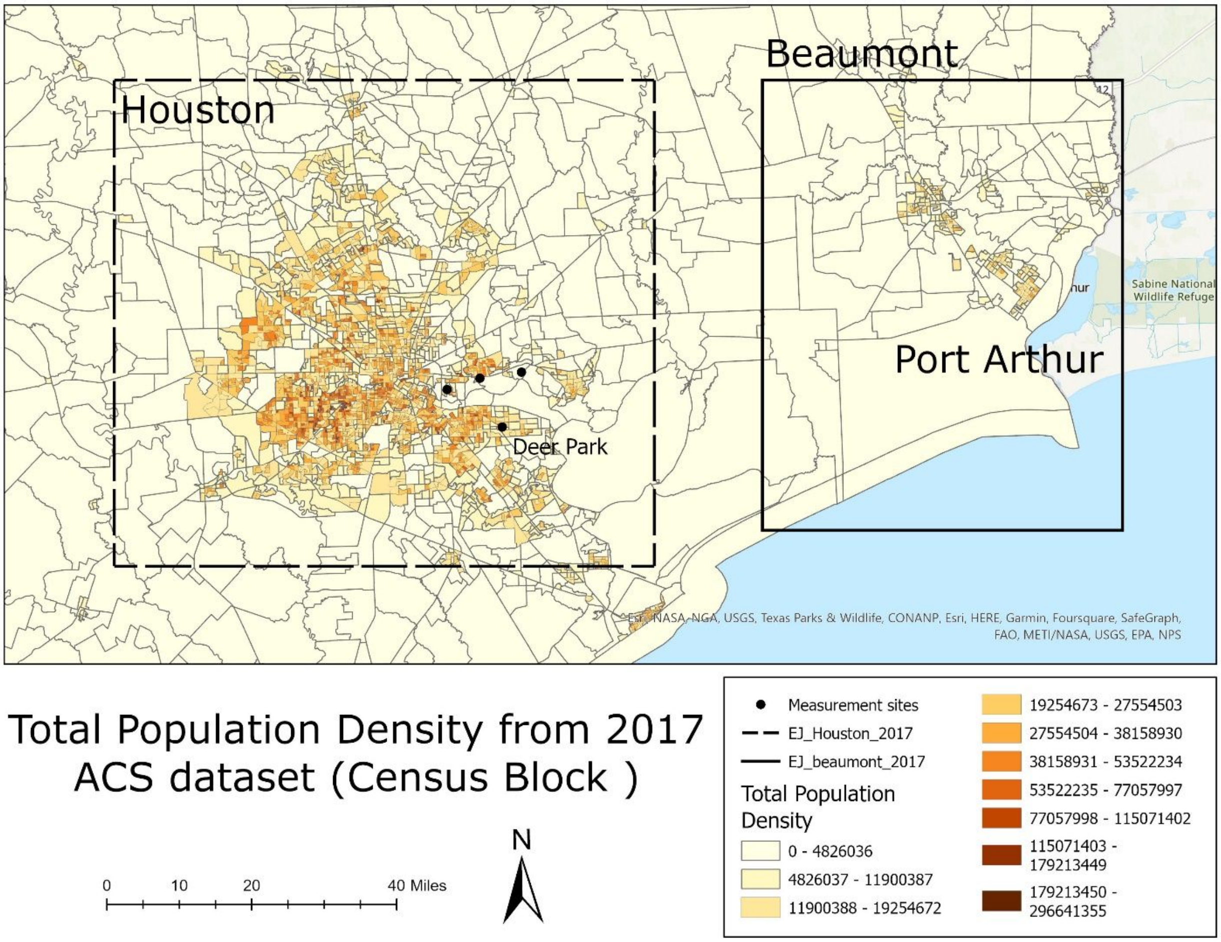

Figure S 3. Total population spatial distribution in target area. Figure created by ArcGIS, licensed by UC Davis.

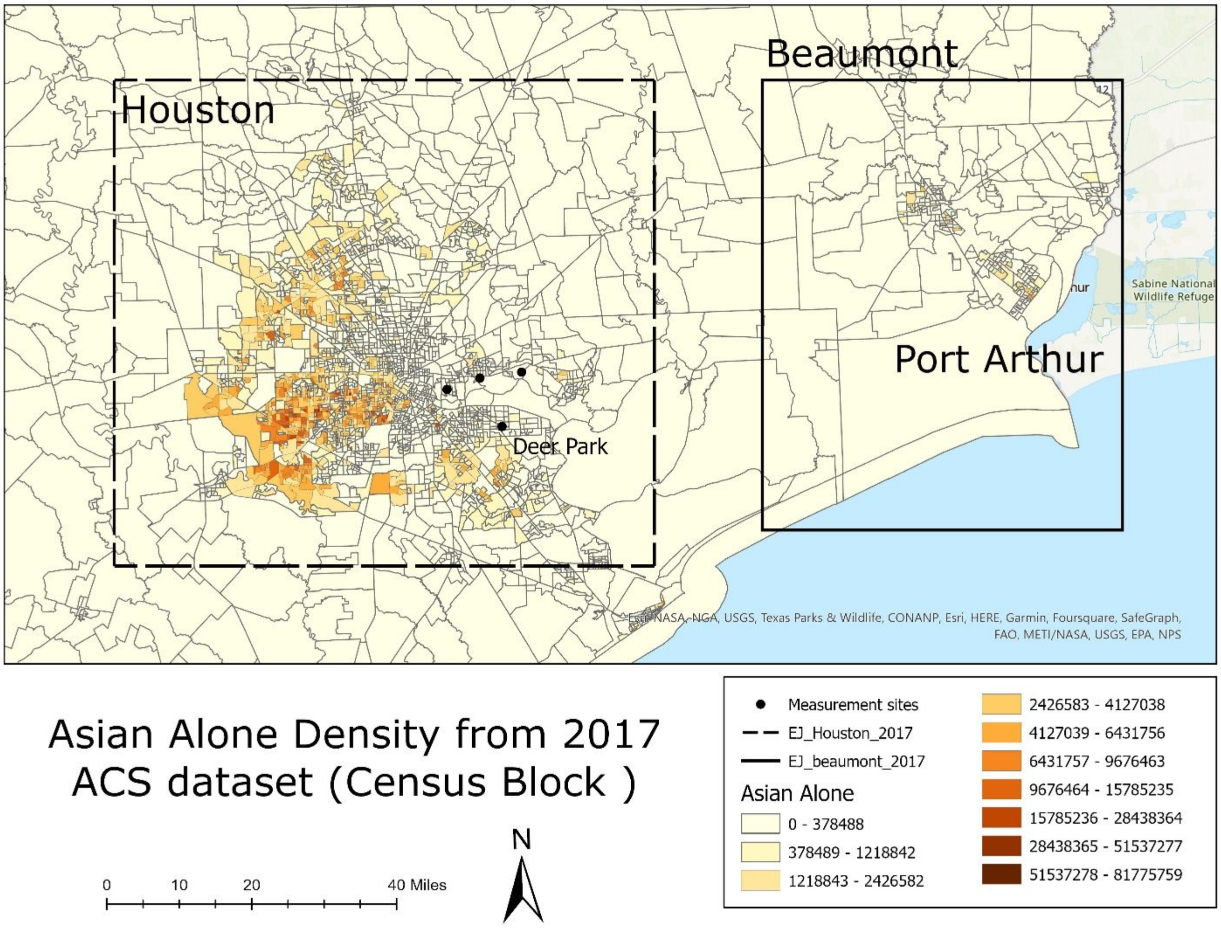

Figure S 4. Asian alone population density in Houston – Beaumont area. Figure created by ArcGIS, licensed by UC Davis.

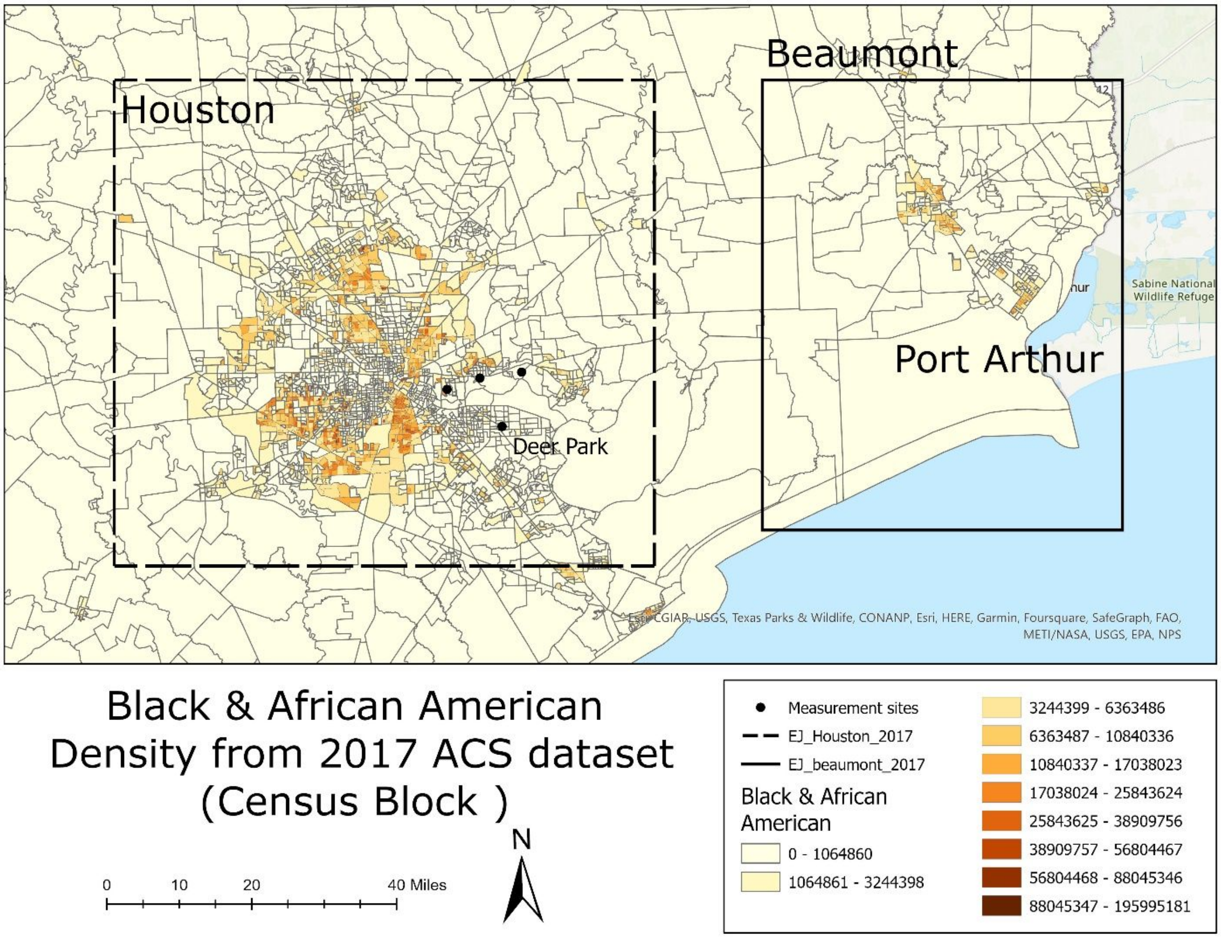

Figure S 5. Black & African American alone population density in Houston – Beaumont area. Figure created by ArcGIS, licensed by UC Davis.

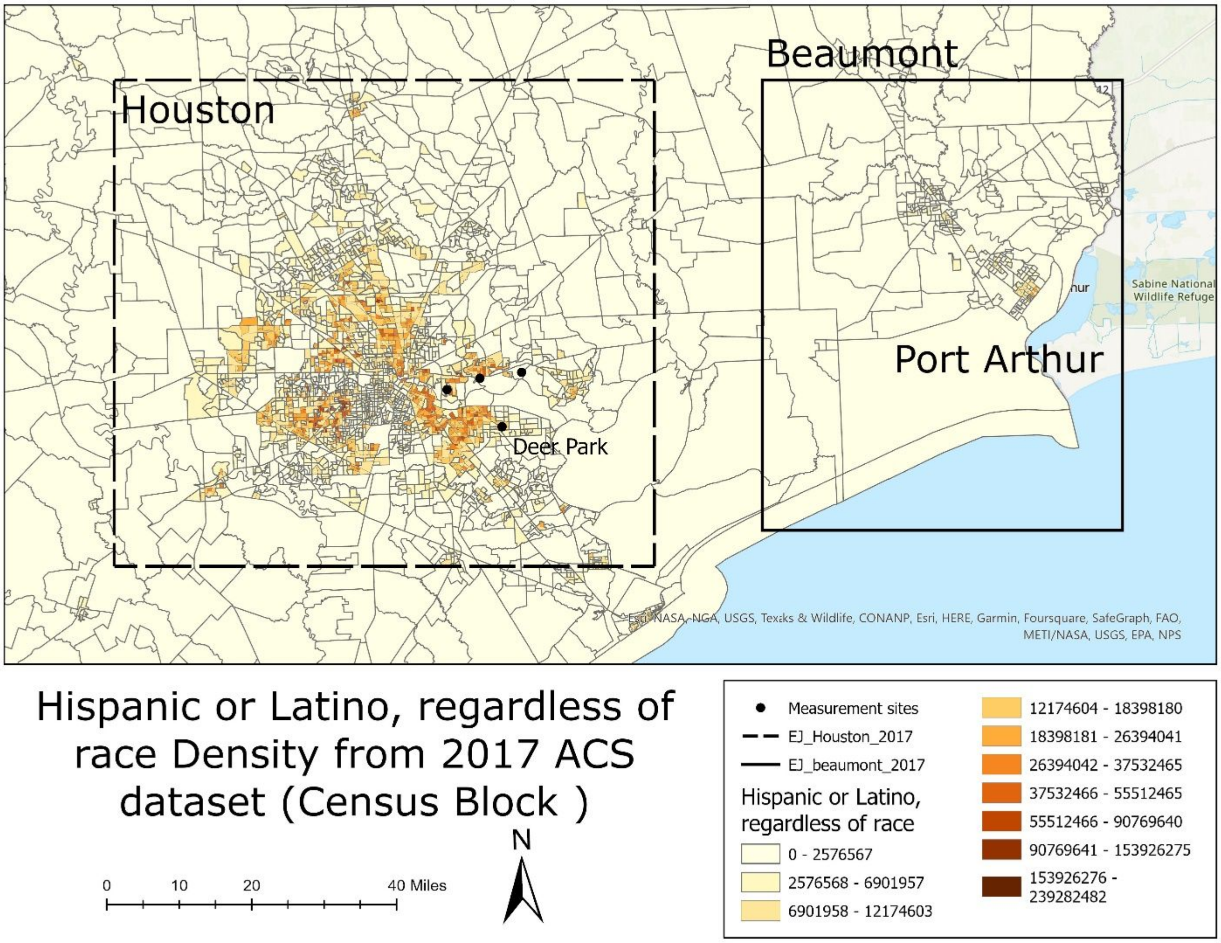

Figure S 6. Hispanic or Latino, regardless of races population density in Houston – Beaumont area. Figure created by ArcGIS, licensed by UC Davis.

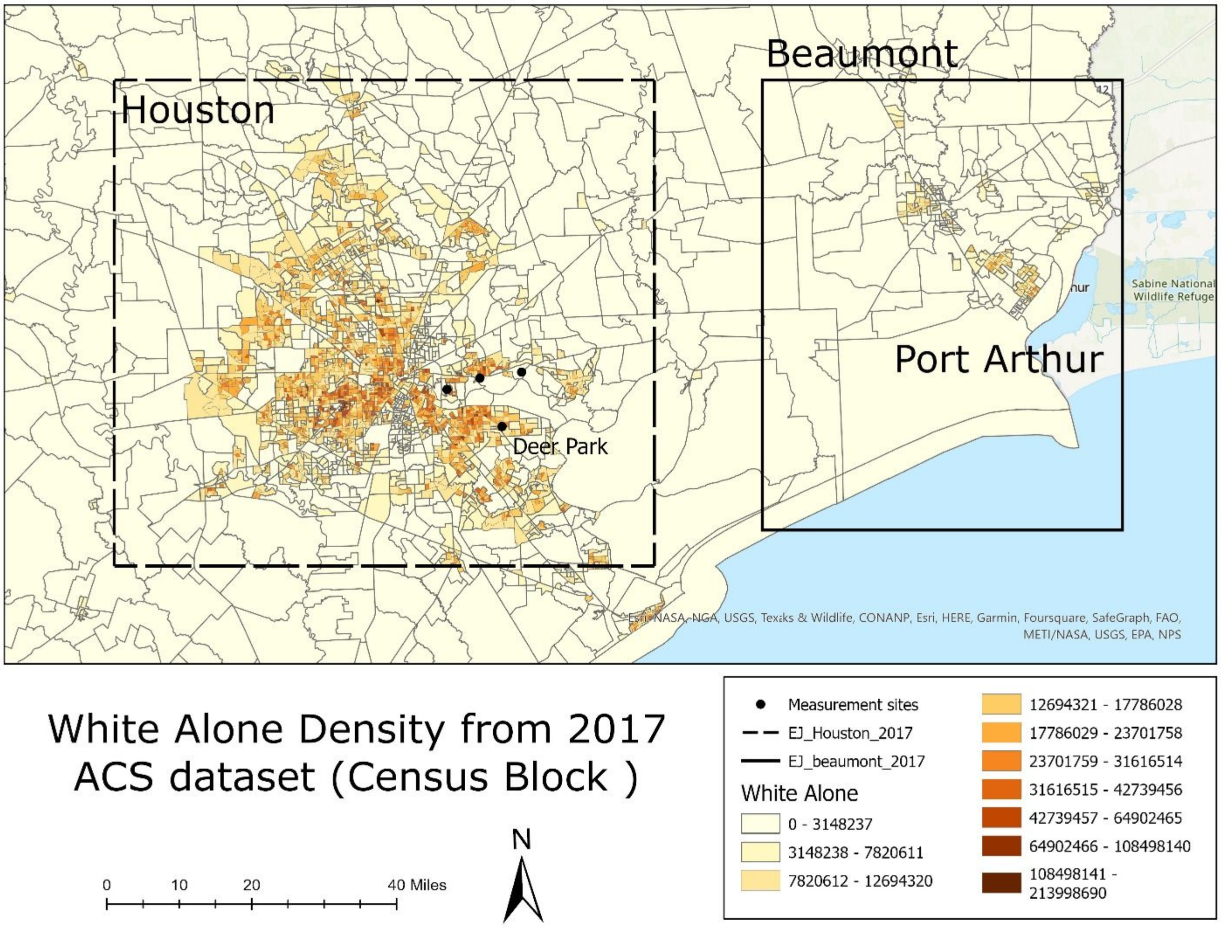

Figure S 7. White alone population density in Houston – Beaumont area. Figure created by ArcGIS, licensed by UC Davis.

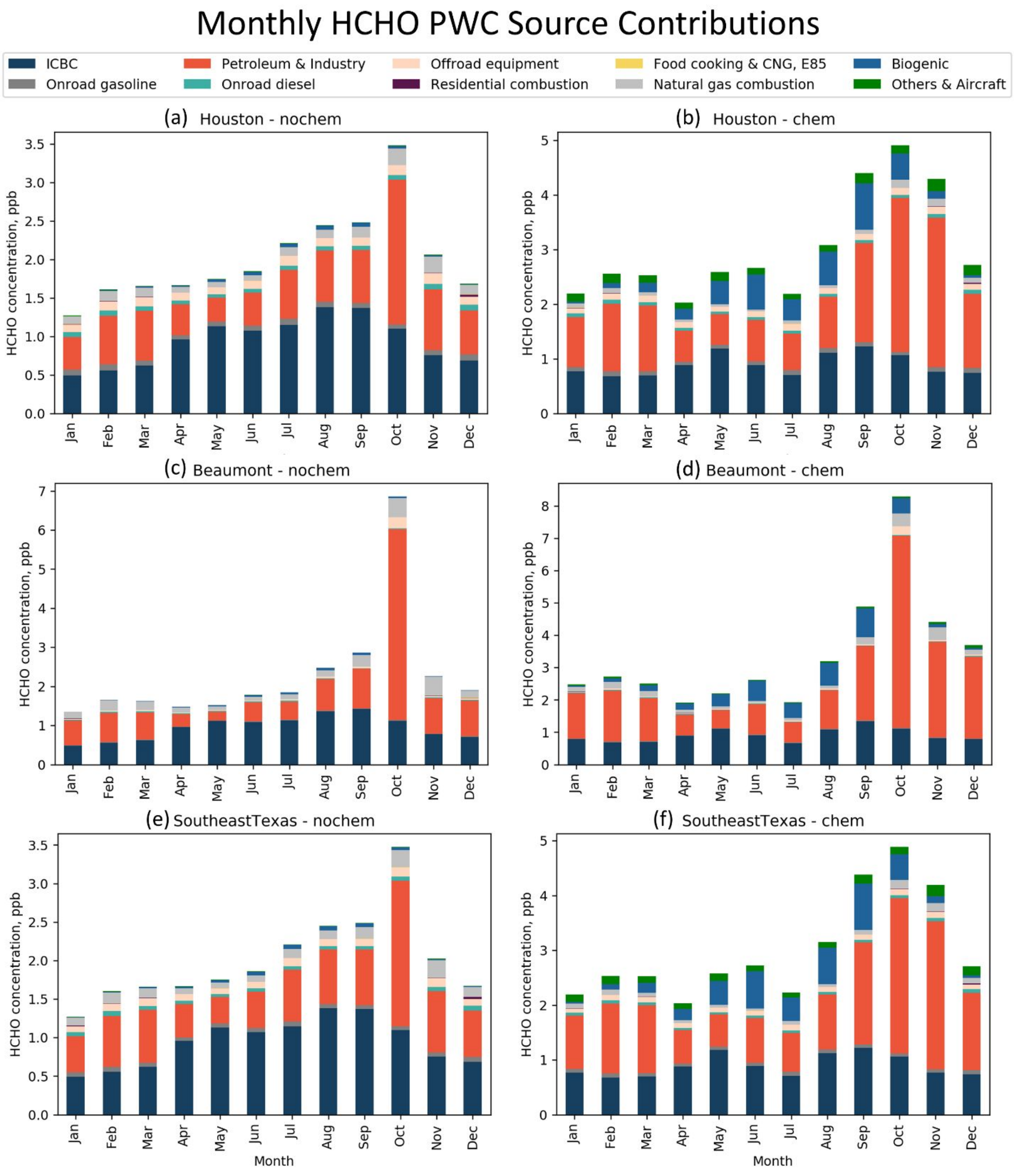

Figure S 8. Monthly HCHO population weighted concentration over Houston EJ domain, Beaumont EJ domain, and Southeast Texas for w/ chem simulation and w/o chem simulation.

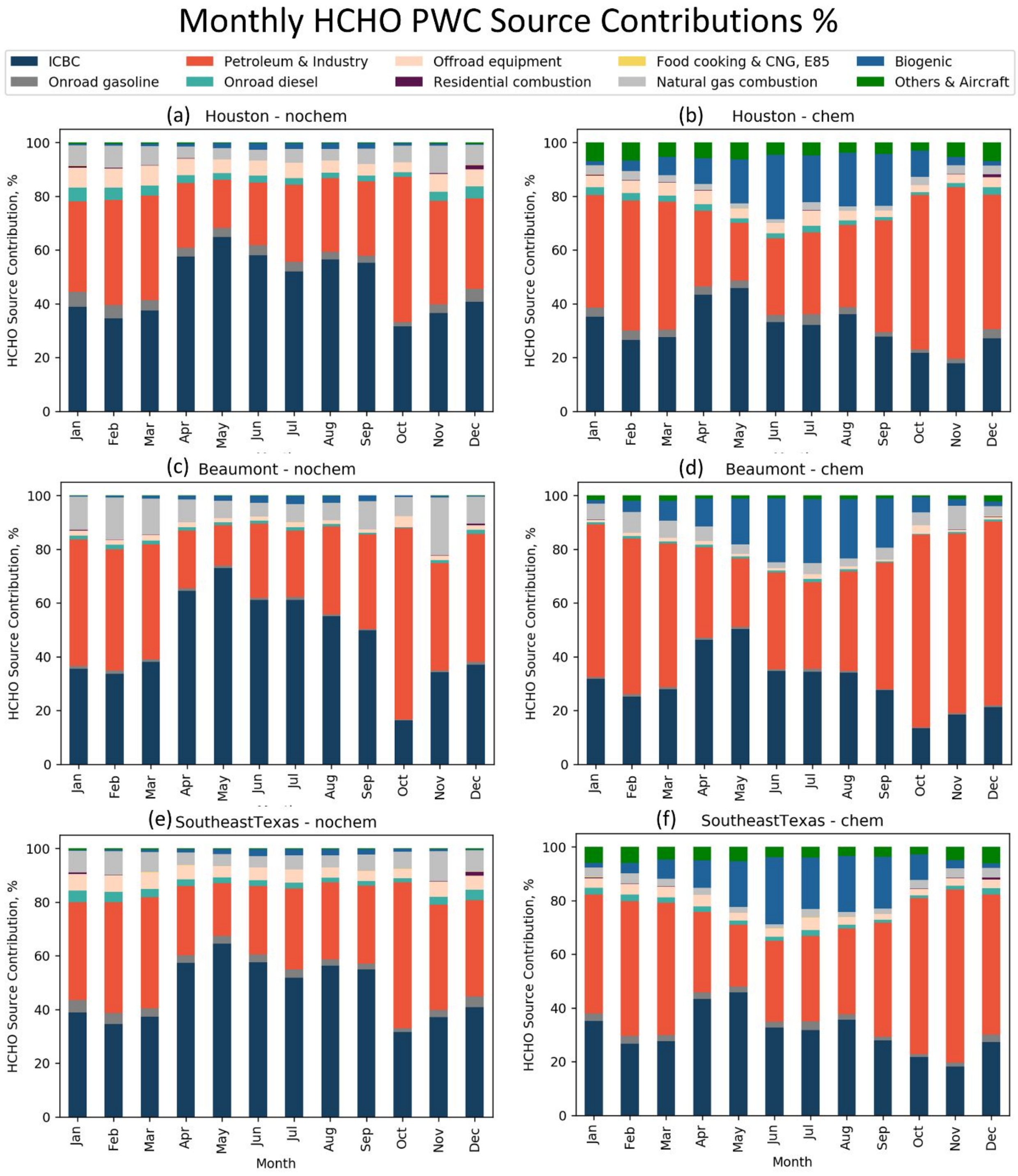

Figure S 9. Monthly HCHO population weighted concentration % over Houston EJ domain, Beaumont EJ domain, and Southeast Texas for w/ chem simulation and w/o chem simulation.

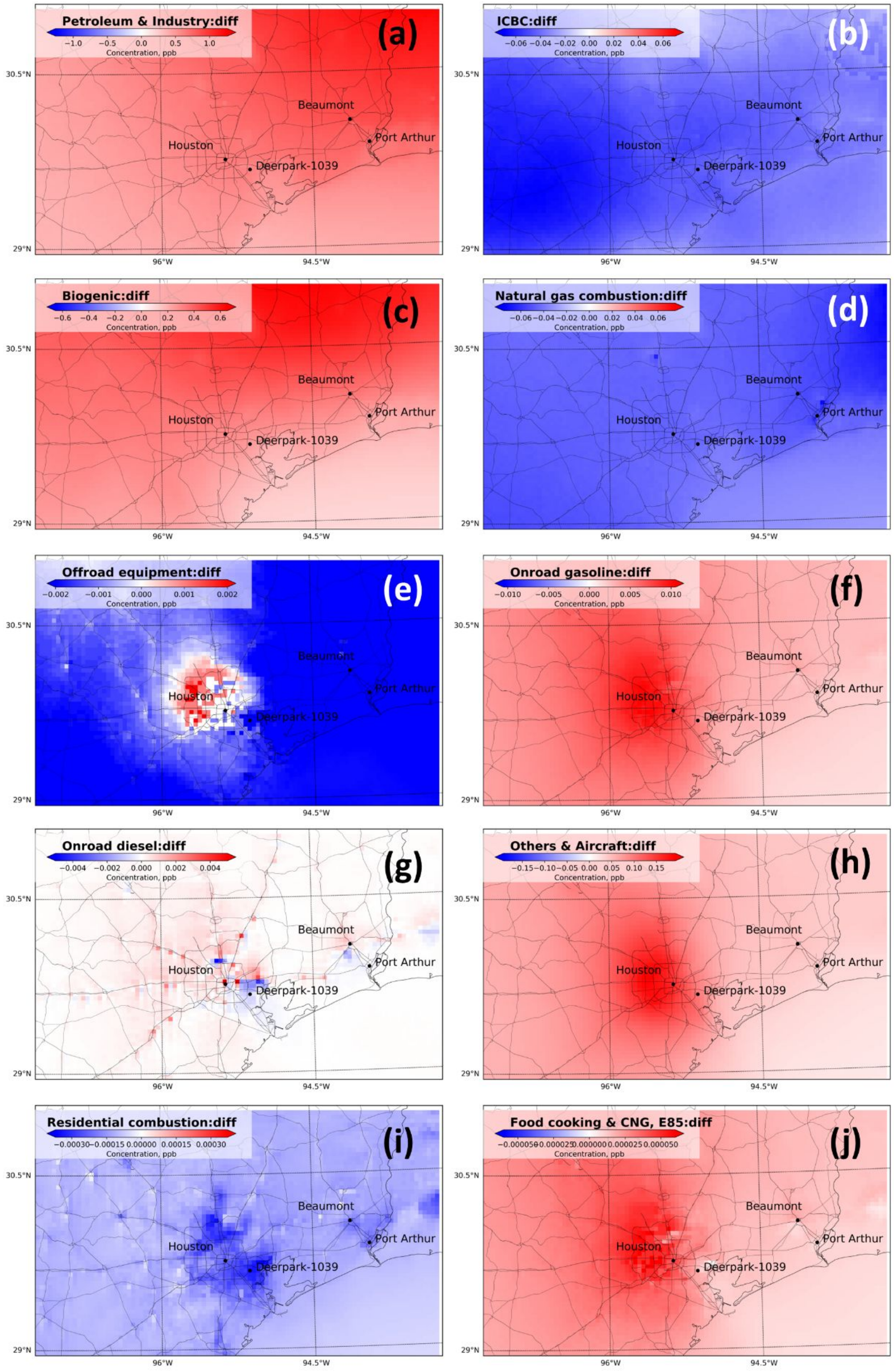

Figure S 10. Secondary HCHO contributions.

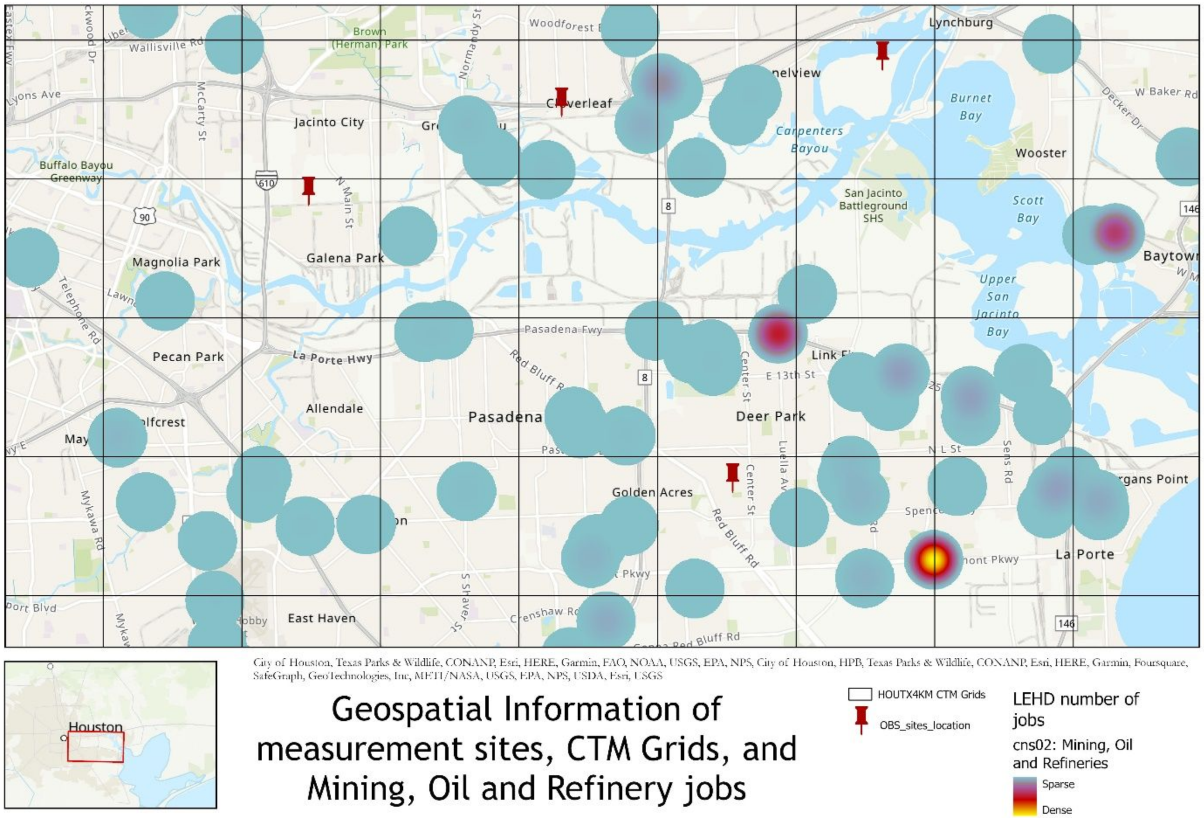

Figure S 11. Measurement sites geospatial information.

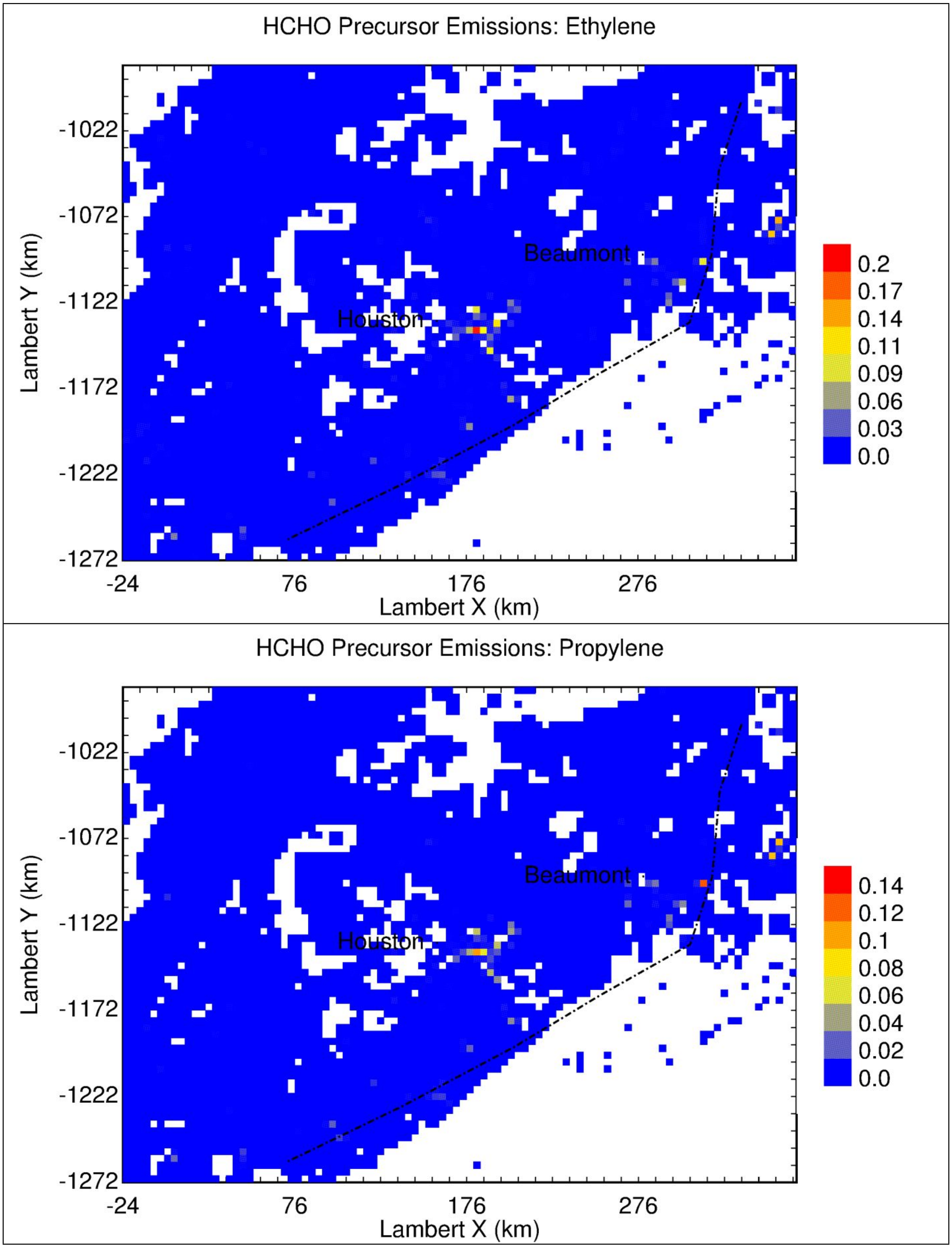

Figure S 12. HCHO precursors emissions – Ethylene and Propylene.

Table S 1. Source type – Petroleum & Industrial top 20 SCC for months except for October.

SCC	Percentage %	Description 1	Description 2	Description 3
31000205	30.88	OIL & GAS PRODN	NATURAL GAS PRODN	FLARES
39999999	10.55	INDUSTRIAL PROCES	MISCELLANEOUS	NOT CLASSIFIED
20200102	9.11	INTERNLCOMBUSTION	INDUSTRIAL	DIST.OIL/DIESEL
30600402	8.12	PETROLEUM INDRY	PETROLEUM REFNG	BLOWDOWN SYSTEM
31000404	5.28	OIL & GAS PRODN	FUEL-FIRED EQPMNT	PROCESS HEATERS
20200202	3.96	INTERNLCOMBUSTION	INDUSTRIAL	NATURAL GAS
30900198	3.67	FABRICATED METALS	IRON/STEEL	NOT CLASSIFIED
10200902	3.48	EXTCOMB BOILER	INDUSTRIAL	WOOD/BARK WASTE
30600999	3.08	PETROLEUM INDRY	PETROLEUM REFNG	FLARES
30600904	3.03	PETROLEUM INDRY	PETROLEUM REFNG	FLARES
10200603	2.46	EXTCOMB BOILER	INDUSTRIAL	NATURAL GAS
20200252	2.19	INTERNLCOMBUSTION	INDUSTRIAL	NATURAL GAS
20200253	2.08	INTERNLCOMBUSTION	INDUSTRIAL	NATURAL GAS
30699999	1.49	PETROLEUM INDRY	MISCELLANEOUS	NOT CLASSIFIED
2310000220	1.25	FUEL COMBUSTION	PETROLEUM PRODUCTIO	WORKOVER RIGS
40899999	0.87	ORGNC CHEM TRNSPT	MISCELLANEOUS	SPECIFY LIQUID
31000415	0.84	OIL & GAS PRODN	FUEL-FIRED EQPMNT	STEAM GENERATORS
10200903	0.7	EXTCOMB BOILER	INDUSTRIAL	WOOD/BARK WASTE
20200201	0.5	INTERNLCOMBUSTION	INDUSTRIAL	NATURAL GAS
30600104	0.5	PETROLEUM INDRY	PETROLEUM REFNG	PROCESS HEATERS
**Total**	**94.04**			

Table S 2. Source type – Petroleum & Industrial top 20 SCC for October only.

SCC	Percentage %	Description 1	Description 2	Description 3
30600201	14.76	PETROLEUM INDRY	PETROLEUM REFNG	CATALYTIC CRACKNG
30600904	14.58	PETROLEUM INDRY	PETROLEUM REFNG	FLARES
30600104	7.59	PETROLEUM INDRY	PETROLEUM REFNG	PROCESS HEATERS
30600999	6.95	PETROLEUM INDRY	PETROLEUM REFNG	FLARES
39999999	6.73	INDUSTRIAL PROCES	MISCELLANEOUS	NOT CLASSIFIED
31000205	6.25	OIL & GAS PRODN	NATURAL GAS PRODN	FLARES
30699999	4.79	PETROLEUM INDRY	MISCELLANEOUS	NOT CLASSIFIED
30600903	3.85	PETROLEUM INDRY	PETROLEUM REFNG	FLARES
30190003	3.47	CHEMICAL MFG	FUEL-FIRED EQPMNT	PROCESS HEATERS
20200102	3.25	INTERNLCOMBUSTION	INDUSTRIAL	DIST.OIL/DIESEL
30600401	2.68	PETROLEUM INDRY	PETROLEUM REFNG	BLOWDOWN SYSTEM
30190099	1.95	CHEMICAL MFG	MISCELLANEOUS	WASTE GAS FLARES
30190004	1.92	CHEMICAL MFG	FUEL-FIRED EQPMNT	PROCESS HEATERS
30600402	1.85	PETROLEUM INDRY	PETROLEUM REFNG	BLOWDOWN SYSTEM
10200701	1.52	EXTCOMB BOILER	INDUSTRIAL	PROCESS GAS
10200799	1.17	EXTCOMB BOILER	INDUSTRIAL	PROCESS GAS
30890023	0.98	Fuel Comb. Industrial	Gas	Natural
30600203	0.97	EXTCOMB BOILER	INDUSTRIAL	PROCESS GAS
30190023	0.89	CHEMICAL MFG	FUEL-FIRED EQPMNT	FLARES
30600905	0.83	PETROLEUM INDRY	PETROLEUM REFNG	FLARES
**total**	**86.98**			

Table S 3. Source type – Offroad equipment top 20 SCC.

SCC	Percentage	Description 1	Description 2	Description 3
2270005022	15.81	Off-Highway	Non-Road Diesel	Farm
2270002022	15.22	Off-Highway	Non-Road Diesel	Construction
2260001022	11.86	Off-Highway	Non-Road Gasoline	Recreational
2285002006	9.58	TRAINS	LOCOMOTIVES	HAULING^M
2265004033	8.48	UTILITY EQUIPMENT	LAWN & GARDEN	RESIDENTIAL
2265006022	7.9	Off-Highway	Non-Road Gasoline	Light Commercial
2265001022	4.58	Off-Highway	Non-Road Gasoline	Recreational
2260004021	4.44	Off-Highway	Non-Road Gasoline	Lawn & Garden
2260004044	3.92	Off-Highway	Non-Road Gasoline	Lawn & Garden
2270006022	3.02	Off-Highway	Non-Road Diesel	Light Commercial
2260004033	2.02	UTILITY EQUIPMENT	LAWN & GARDEN	RESIDENTIAL
2260002022	1.84	Off-Highway	Non-Road Gasoline	Construction
2265004044	1.71	UTILITY EQUIPMENT	LAWN & GARDEN	COMMERCIAL
2265004022	1.27	Off-Highway	Non-Road Gasoline	Lawn & Garden
2260006022	1.14	Off-Highway	Non-Road Gasoline	Light Commercial
2260004020	0.88	Off-Highway	Non-Road Gasoline	Lawn & Garden
2270003022	0.86	Off-Highway	Non-Road Diesel	Industrial
2265002022	0.84	Off-Highway	Non-Road Gasoline	Construction
2270003060	0.8	Off-Highway	Non-Road Diesel	Industrial
2265005022	0.64	Off-Highway	Non-Road Gasoline	Farm
**Total**	**96.81**			

Table S 4. Source type – natural gas combustion top 19 SCC.

SCC	Percentage	Description 1	Description 2	Description 3
10200602	28.71	EXTCOMB BOILER	INDUSTRIAL	NATURAL GAS
20200254	18.1	INTERNLCOMBUSTION	INDUSTRIAL	NATURAL GAS
10100601	16.34	EXTCOMB BOILER	ELECTRIC GENERATN	NATURAL GAS
10100604	12.24	EXTCOMB BOILER	ELECTRIC GENERATN	NATURAL GAS
10200604	6.33	EXTCOMB BOILER	INDUSTRIAL	NATURAL GAS
20100201	4.9	INTERNLCOMBUSTION	ELECTRIC GENERATN	NATURAL GAS
10200601	4.69	EXTCOMB BOILER	INDUSTRIAL	NATURAL GAS
2104006000	2.82	FUEL COMBUSTION	RESIDENTIAL	NAT GAS UNSPECIFIE
10200603	1.72	EXTCOMB BOILER	INDUSTRIAL	NATURAL GAS
20200201	1.15	INTERNLCOMBUSTION	INDUSTRIAL	NATURAL GAS
2103006000	0.95	FUEL COMBUSTION	OTHER	CO-GENERATION^M
20200203	0.85	INTERNLCOMBUSTION	INDUSTRIAL	NATURAL GAS
20300203	0.31	INTERNLCOMBUSTION	COMMERCL-INSTUTNL	NATURAL GAS
2268006022	0.27	Off-Highway	Other	Compressed Natural
2104007000	0.21	FUEL COMBUSTION	RESIDENTIAL	L.P.G
2102006000	0.21	FUEL COMBUSTION	INDUSTRL IC ENGINES	NATURAL GAS
2268003022	0.2	Off-Highway	Other	Compressed Natural
**Total**	**100**			

Table S 5. Source type – other & aircraft top 11 SCC.

SCC	Percentage	Description 1	Description 2	Description 3
2267003022	75.55	Off-Highway	Industrial equip – Aerial Lifts	Liquified Petroleum gas
2267006022	10.26	Off-Highway	Agricultural	Liquified Petroleum gas
2460500000	6.47	SOLVENT USE	CONSUMER PRODUCTS	METAL POLISH/CLEANS
2103007000	3.28	FUEL COMBUSTION	COMMERCIAL	L.P.G
2102007000	2.79	FUEL COMBUSTION	INDUSTRIAL	L.P.G
30100899	0.52	CHEMICAL MFG	INORGANIC CHEMCLS	CHLORO-ALKALI
30199998	0.36	CHEMICAL MFG	MISCELLANEOUS	NOT CLASSIFIED
2461850000	0.35	PESTICIDE USE	UNSPECIFIED PEST	CREOSOTE APPLICATN
2268005022	0.2	INDUSTRIAL EQUIPMENT	LIGHY DUTY	L.P.G.
2267004044	0.05	INDUSTRIAL EQUIPMENT	LIGHY DUTY	L.P.G.
10101206	0.04	Fuel Comb. Elec. Util.	Other	Other
**Total**	**99.87**			
